# Omic insights into various ceftazidime-avibactam-resistant *Klebsiella pneumoniae* isolates from two southern Italian regions

**DOI:** 10.3389/fcimb.2022.1010979

**Published:** 2023-01-05

**Authors:** Dafne Bongiorno, Dalida A. Bivona, Claudia Cicino, Enrico M. Trecarichi, Alessandro Russo, Nadia Marascio, Maria Lina Mezzatesta, Nicolò Musso, Grete F. Privitera, Angela Quirino, Giuseppe G. M. Scarlata, Giovanni Matera, Carlo Torti, Stefania Stefani

**Affiliations:** ^1^ Microbiology Section, Dept of Biomedical and Biotechnological Science, University of Catania, Catania, Italy; ^2^ Unit of Clinical Microbiology, Department of Health Sciences, “Magna Graecia” University, Catanzaro, Italy; ^3^ Unit of Infectious and Tropical Diseases, Department of Medical and Surgical Sciences, “Magna Graecia” University, Catanzaro, Italy; ^4^ Unità Operativa Complessa (UOC) Laboratory Analysis, University Hospital Policlinico-San Marco, Catania, Italy

**Keywords:** next generation sequencing, KPC, OmpK proteins, resistome, virulome

## Abstract

Ceftazidime-avibactam (CZA) is one of the best therapeutic options available for infections caused by *Klebsiella pneumoniae* carbapenemase (KPC)-producing bacteria. However, sporadic reports of CZA-resistant strains have been rapidly increasing in patients. Herein, we provide detailed case reports of the emergence of ceftazidime-avibactam resistance to identify their resistome and virulome using genomic molecular approaches. Sixteen isolates were collected from 13 patients at three hospitals in Catania and Catanzaro (Italy) between 2020-2021. Antimicrobial susceptibility was determined by broth microdiluition. The samples included in study were analyzed for resistome, virulome and Sequence Type (ST) using Whole Genome Sequencing (WGS). All strains were resistant to ceftazidime/avibactam, ciprofloxacin, extended-spectrum cephalosporins and aztreonam, 13/16 to meropenem, 8/16 to colistin and 7/16 to fosfomycin; 15/16 were susceptible to meropenem/vaborbactam; all strains were susceptible to cefiderocol. Molecular analysis showed circulation of three major clones: ST101, ST307 and ST512. In 10/16 strains, we found a *bla*_KPC-3_ gene; in 6/16 strains, four different *bla*_KPC_ variants (*bla*_KPC28-31-34-50_) were detected. A plethora of other beta-lactam genes (*bla*_SHV28-45-55-100-106-187-205-212_, *bla*_OXA1-9-48_, *bla*_TEM-181_ and *bla*_CTX-M-15_) was observed; *bla*_OXA-9_ was found in ST307 and ST512, instead *bla*_OXA48_ in one out four ST101 strains. With regard to membrane permeability, *omp*K35 and *omp*K36 harbored frameshift mutations in 15/16 strains; analysis of *omp*K37 gene revealed that all strains harbored a non-functional protein and carry wild-type PBP3. There is an urgent need to characterize the mechanisms underlying carbapenem resistance and the intrinsic bacterial factors that facilitate the rapid emergence of resistance. Furthermore, it is becoming increasingly important to explore feasible methods for accurate detection of different KPC enzymes.

## Introduction

For KPC-producing *Enterobacterales* are categorized as one of the most dangerous pandemics in the history of Gram-negative bacteria, particularly due to *Klebsiella pneumoniae* ST258 and variants (Patel and Bonomo, 2013).These strains have been reported in many countries, including Italy (Giani et al., 2013; Gona et al., 2014), where the majority of CZA-resistant KPC-producing *K. pneumoniae* were observed in the sequence type 512 (ST512) multi-drug resistance (MDR) clone, with some recent reports of ST101 and ST307 as well (Carattoli et al., 2021).

Currently, ceftazidime/avibactam (CZA), a combination of a third-generation, broad-spectrum cephalosporin and a β-lactamase inhibitor that inactivates ESBLs (TEM, SHV, CTX-M), *Amp*C and carbapenemases (KPC-2, KPC-3, OXA-10 and OXA-48), but not metallo-enzymes (Shirley, 2018), is one of the best therapeutic options available for infections caused by such isolates. However, emergence of CZA resistance in carbapenemase-producing *Enterobacterales* (CPE) has been reported in patients (Shields et al., 2017; Giddins et al., 2018). Several mechanisms of CZA resistance have been linked to specific mutations in the *bla*_KPC_ gene (Shields et al., 2017; Giddins et al., 2018) and to permeability defects (porin deficiency), often combined with increased expression of KPC or ESBL determinants (Humphries and Hemarajata, 2017).

From a clinical standpoint, it is important to understand the cross-resistance pattern of these microorganisms in order to optimize drug selection and guide treatment strategies. In this sense, it is mandatory to characterize the genes involved in phenotypic and clinical resistance by means of rapid tests.

In this study, we described the characterization of CZA-resistant KPC-producing *K. pneumoniae* strains isolated in three different hospitals. The great majority of these strains retained activity to meropenem/vaborbactam, while all strains were susceptible to cefiderocol. Three different ST-types were identified, and whole genome sequencing (WGS) analysis revealed differences in CZA resistance mechanisms resulting from the complex resistome and virulome profiles of these isolates.

## Materials and methods

### Sample

The present study was conducted at three hospitals located in Catania (A.O.U. Policlinico “G. Rodolico - San Marco” and “A.O. Cannizzaro”) and Catanzaro (“Mater Domini” University Hospital of the “Magna Graecia” University) in the period between May 2020 and October 2021. Included were all CZA-resistant KPC-producing *K. pneumoniae* isolates collected from each patient. For a same patient, more than one strain was included if isolated from two different clinical samples collected at least two weeks apart from each other. Identification and antimicrobial susceptibility had been previously performed by the Vitek 2 system (bioMerieux, Marcy l’Etoile, France) at the above mentioned hospitals and re-confirmed by standard methods (EUCAST, 2022).

In addition, minimum inhibitory concentrations (MICs) were performed at the microbiology laboratories involved, using broth microdilution as described by the CLSI guidelines (Clinical and Laboratory Standards Institute, 2015). The following antibiotics were tested: ceftazidime/avibactam (CZA), amoxicillin/clavulanate (AMC), piperacillin/tazobactam (TZP), cefepime (FEP), ceftazidime (CAZ), ceftazolane/tazobactam (C/T), meropenem (MEM), imipenem (IMI), aztreonam (AZT), meropenem/vaporbactam (MEV), cefiderocol (FDC), ciprofloxacin (CIP), tobramycin (TOB), amikacin (AK), gentamycin (CN), colistin (CS), fosfomycin (FOS), trimethoprim/sulfamethoxazole (SXT). Breakpoints of antibiotics for the interpretative criteria for clinical isolates were used according to the EUCAST v 12.0 (EUCAST, 2022). *E. coli* ATCC 25922 was used as the quality control strain. Sample characteristics, date of isolation, patient demographics and therpy are reported in **Table S1**.

### DNA extraction

DNA extraction was carried out following the manufacturer’s instructions provided by QIAGEN QIAamp^®^ DNA Mini Kit (Ref. 51304, QIAGEN, 40724 Hilden, Germany).

DNA was quantified using both the Eppendorf BioPhotometer^®^ D30 and the fluorimeter Qubit dsDNA BR Assay Kit to evaluate purity and quantity of the initial sample, respectively (Ref. 32850, Invitrogen, 92008 Carlsbad, CA, USA). The results of quantification are shown in **Table S2**.

### NGS Sequencing

A concentration of 100 ng of each sample was used for NGS sequencing. This was performed at the Molecular Biology laboratory of University of Catania on a Illumina MiSeq platform according to the manufacturer’s instructions provided in the Illumina DNA Prep – (M) Tagmentation for Illumina^®^ (Ref. 20018707, Illumina, Inc., 92122, San Diego, CA, USA). Indexes were provided with Nextera™ DNA CD Indexes Illumina^®^ (24 Indexes, 24 Samples) (Ref. 20019105, Illumina, Inc., 92122, San Diego, CA, USA).

Libraries were quantified and their quality evaluated using both the fluorometric Qubit dsDNA HS Assay Kit (Ref. Q32851, Invitrogen, Carlsbad, CA 92008, USA) and the Agilent^®^ High Sensitivity DNA Kit (Ref. 5067-4626).

Denature and dilute libraries were performed following the “Denature and Dilute Libraries Guide” protocol provided by Illumina^®^, choosing 8,5 pM as the loading concentration. Finally, sequencing was performed using the MiSeq Reagent Kits v3 (Ref. 15043895, Illumina, Inc., 92122, San Diego, CA, USA). The Sample Sheet was created using the Local Run Manager v3 software, and following the instructions in the Local Run Manager v3 Software Guide provided by Illumina (Software Guide provided by Illumina (Local run manager generate FASTQ analysis module workflow guide, 2018)).

### Data analysis

Data were analyzed using the QIAGEN CLC Genomics Workbench software and following the User Manual for the *CLC Microbial Genomics Module v22.0*, released on January 4, 2022 (QIAGEN, Aarhus, 8000 Denmark), that uses the CARD database to assign resistance, virulence and MLST genes (https://card.mcmaster.ca/).

### Bioinformatic analysis

Sixteen paired-end bacterial raw reads were firstly trimmed with TrimGalore (v0.5.0) (Martin, 2011; Krueger, 2022) to remove the adapter sequence. After that, bacterial genome was assembled *de novo* using Unicycler (v0.4.8) (Wick et al., 2017) with the Illumina only assembly modality. Known virulence factors, resistance genes and capsule loci were identified using Kleborate (v2.2.0) and the Kaptive command (Wyres et al., 2016; Lam et al., 2021). Prokka (v1.13) (Seemann, 2014) was used for bacterial annotation. Moreover, Unicycler output assemblies were aligned with several protein sequences in order to identify punctual mutations.

Thereafter, files were sorted by SAM tools, variants were called and consensus sequences were generated using BCTftools (Li, 2011; Danecek et al., 2021).

### Statistical analysis

In order to establish a potential correlation between beta-lactams MIC values and related genes, Pearson’s correlation analyses were performed for the 16 isolates. This correlation matrix is a statistical tool that measures the linear correlation between two variables, X and Y. It has a value between +1 and −1, where +1 indicates total positive linear correlation, 0 no linear correlation, and −1 total negative linear correlation. The correlation coefficient ranges from −1 to 1, where 1 implies that a linear equation perfectly describes the relationship between X and Y, with all data points lying on a line for which Y increases as X increases. In addition to this, R-Squared was also calculated. This measures the reliability of the linear relationship between the variables included in the model. Its value is between 0 (fully correlated variables) and 1 (unrelated variables).

For all statistical comparisons, a significance level of p <0.05 was considered to show differences between the groups. Data analysis was performed using Prims Version 9.4.0, June 3, 2022.

## Results

### Clone characteristics

A total of 16 CZA-resistant KPC-producing *K. pneumoniae* strains were isolated from clinical samples collected from 13 patients and included in the study (**Table S1**, **Supplementary Material 1**). They were MDR and resistant to CZA, with MICs one or two dilutions higher than the clinical breakpoint.

Isolates 1/2CT, 6/7CZ, 8/9CZ were collected from the same patients. In 1/2CT and 6/7CZ patients have not been treated with CZA during the previous 12 months (**Table S1**). All strains were also resistant to ciprofloxacin, extended-spectrum cephalosporins and aztreonam, 13/16 were resistant to meropenem, while 15/16 (MIC range of 4->32 mg/L) were susceptible to meropenem/vaborbactam (MIC range 0.006-0.5 mg/L); all strains were fully susceptible to cefiderocol (MIC range 0.006-0.5 mg/L). With regard to colistin and fosfomycin, resistance was observed in 8/16 and 7/16 strains, respectively.

All strains were genomically identified as belonging to three different STs, namely ST101, 307 and 512, whereas the LPS O-antigen, composed of D-galactans, was encoded by genes that differentiate between two serotypes, i.e., O1/O2v1 and O1/O2v2, distinguished by rearrangements detected in the *rfb* region, and possessed three different capsular polysaccharides (*K* locus) K17, 102, 107 linked to the *wzi* gene sequence (**Table 1**).

**Table 1:** Phenotypic and Molecular Characterization and Resistance Profile of the *K. pneumonia*.

							Beta-lactams											Fluoro chinolone	Amino glycoside			Colis tine	Fosfo mycin	Sulfo namide
	Mount/Year	Source	MLST	wzi	K-locus	O-locus	CZA	AMC	TZP	FEP	CAZ	C/T	MEM	IMI	AZT	MEV	FDC	CIP	TOB	AK	CN	CS	FOS	SXT
							S≤8	S≤8	S≤8	S≤1	S≤1	S≤2	S≤2	S≤2	S≤1	S≤8	S≤2	S≤0,25	S≤2	S≤8	S≤2	S≤2	S≤32	S≤2
							R>8	R>8	R>8	R>4	R>4	R>2	R>2	R>4	R>4	R>8	R>2	R>0,5	R>2	R>8	R>2	R>2	R>32	R>4
**1CT**	**06/20**		ST101	137	K17	O1/O2v1	16	32	>64	32	>32	>256	1	≤0,25	32	0,5	1	>2	16	32	16	≤0,25	16	2
**2CT**	**06/20**		ST101	137	K17	O1/O2v1	16	>32	64	16	>32	>256	16	16	>1024	2	0,5	2	8	32	8	≤0,25	64	0,75
**3CT**	**05/20**		ST101	137	K17	O1/O2v1	16	32	>64	32	>32	>256	1	≤0,25	>1024	0,5	0,5	>2	16	32	16	≤0,25	128	3
**4CT**	**08/21**		ST101	137	K17	O1/O2v1	16	32	>64	32	>32	32	16	16	1024	64	0,064	>2	16	>256	16	≤0,25	128	356
**5CT**	**10/21**		ST101	137	K17	O1/O2v1	16	16	16	32	>32	32	2	≤0,25	6	0,25	0,5	>2	16	4	16	16	128	≤2
**1CZ**	**04/20**		ST307	173	K102	O1/O2v2	16	>32	32	32	16	8	16	8	8	0,06	0,064	2	8	4	8	16	128	16
**2CZ**	**05/20**		ST512	154	K107	O1/O2v2	16	>32	32	16	16	8	>32	16	8	2	0,25	2	8	16	≤2	8	32	16
**3CZ**	**06/20**		ST512	154	K107	O1/O2v2	16	>32	32	>32	16	8	16	16	8	0,25	0,125	2	8	32	≤2	8	32	16
**4CZ**	**06/20**		ST307	173	K102	O1/O2v2	16	>32	32	16	16	8	>32	16	8	0,5	0,006	2	≤2	4	≤2	1	≤16	16
**5CZ**	**08/20**		ST512	154	K107	O1/O2v2	32	>32	64	32	32	32	4	0,5	8	1	1	4	8	16	8	≤0,25	≤16	16
**6CZ**	**08/20**		ST512	154	K107	O1/O2v2	16	>32	32	16	16	8	>32	16	8	1	0,125	2	8	16	≤2	16	≤16	8
**7CZ**	**09/20**		ST512	154	K107	O1/O2v2	16	>32	32	16	16	8	>32	16	8	1	0,125	2	8	16	≤2	8	128	8
**8CZ**	**08/20**		ST512	154	K107	O1/O2v2	32	>32	32	16	32	8	>32	16	8	0,5	0,25	2	8	32	≤2	1	≤16	512
**9CZ**	**09/20**		ST512	154	K107	O1/O2v2	32	>32	32	32	32	8	16	32	8	0,5	0,25	2	8	32	8	16	≤16	512
**10CZ**	**12/20**		ST101	137	K17	O1/O2v1	32	>32	64	>32	32	32	16	8	32	0,06	1	4	≥16	32	8	≤0,25	4	≤1
**12CZ**	**02/21**		ST307	173	K102	O1/O2v2	32	>32	>64	>32	32	64	32	>32	64	0,25	0,012	4	≥16	16	16	4	64	16

Antibiotic tested: CZA, ceftazidime/avibactam; AMC, amoxicillin/clavulanate; TZP, piperacillin/tazobactam; FEP, cefepime; CAZ, ceftazidime; C/T, ceftazolane/tazobactam; MEM- meropenem; IMI, imipenem; AZT, aztreonam; MEV- meropenem/vaporbactam ; FDC, cefiderocol; CIP, ciprofloxacin; TOB, tobramycin; AK, amikacin; CN, gentamycin; CS, colistin; FOS, fosfomycin; SXT,trimethoprim/sulfamethoxazole.

⬤ Bronchoalveolar lavage.

⬤ Rectal swab.

⬤ Blood culture.

⬤ troath swab.

⬤ Burn swab.

### Resistome analyses

A common core of resistance genes was detected in all isolates, namely: *amp*H (*E. coli* beta-lactamase), *kpn*E-F-G (*K. pneumoniae* efflux pump), *oqx*A-B (quinolone - efflux pump), *lpt*D (LPS assembly protein – efflux pump), *mdf*A (*E. coli* quinolone - efflux pump), *arn*T and *ept*B (altering cell wall), and *omp*A (protein modulating permeability to antibiotics).

Furthermore, the 16 CZA-resistant strains revealed, together with *bla*_KPC_ genes, the constant presence of different *bla*_SHV_ alleles (*bla*_SHV-28-45-55-100-106-187-205-212_) and different *bla*_OXA_ and *bla*_TEM_ genes in their resistome profile. Regarding acquired resistome, different *bla*_KPC_ variants were detected in all isolates, namely 3, 28, 31, 34; *bla*_KPC-3_ in 10/16 isolates, *bla*_KPC-31_ in 3/16, *bla*_KPC-28_, *bla*_KPC-34_ and *bla*_KPC-50_ in one isolate (**Table 2**).

**Table 2:** Resistance profile and gene associated.

						Beta-lactams											Fluorochinolone	Aminoglycoside			Colistine	Fosfomycin	Sulfonamide	Beta-Lactamase																	Fluorochinolone		Aminoglycoside											Fosfomycin	Sulfonamide					phenicoles	Macrolides Eritromycin Tetracicline
	Year	MLST	wzi	K-locus	O-locus	CZA	AMC	TZP	FEP	CAZ	C/T	MEM	IMI	AZT	MEV	FDC	CIP	TOB	AK	GM	CS	FOS	SXT	SHV-28	SHV-45	SHV-55	SHV-100	SHV-106	SHV-187	SHV-205	SHV-212	KPC-3	KPC-28	KPC-31	KPC-34	KPC-50	OXA-1	OXA-9	TEM-181	CTX-M-15	QnrS1	QnrB17	APH(3')-Ia	APH(3'')-Ib	APH(3'')-Ib10	APH(6)-Id	AAC(3)-IIe	AAC(6')-Ib-cr6	AAC(6')-Ib10	aadA2	ANT(2'')-Ia	ANT(3'')-IIa	ArmA	FosA6	sul1	sul2	dfrA12	dfrA14	qacEdelta1	catB3	catImphA mphE msrE tet(A)
**1CT**	**2020**	ST101	137	K17	O1/O2v1					** **												** **		**1**	**0**	**0**	1	0	0	0	0	0	0	1	0	0	0	0	0	0	0	0	0	0	0	0	0	0	0	0	1	1	1	0	1	0	0	0	1	0	10 1 1 0
**2CT**	**2020**	ST101	137	K17	O1/O2v1					** **												** **		**0**	**0**	**0**	0	0	0	0	0	0	0	0	1	0	0	0	0	0	0	0	0	0	0	0	0	0	0	0	1	1	1	0	1	0	0	0	1	0	10 1 1 0
**3CT**	**2020**	ST101	137	K17	O1/O2v1					** **												** **		**1**	**0**	**0**	1	0	0	0	0	0	1	0	0	0	0	0	0	0	0	0	0	0	0	0	0	0	0	0	1	1	1	0	1	0	0	0	1	0	10 1 1 0
**4CT**	**2021**	ST101	137	K17	O1/O2v1					** **												** **		0	0	0	0	0	1	0	1	1	0	0	0	0	0	0	0	0	0	0	0	0	0	0	0	0	0	0	1	1	1	0	1	0	0	0	1	0	10 1 1 0
**5CT**	**2021**	ST101	137	K17	O1/O2v1					** **												** **		**0**	**1**	**1**	0	0	0	0	0	1	0	0	0	0	0	0	0	0	0	0	1	0	0	0	0	0	0	0	0	0	0	0	1	0	0	0	1	0	00 0 0 0
**1CZ**	**2020**	ST307	173	K102	O1/O2v2					** **												** **		1	0	0	0	1	1	1	0	1	0	0	0	0	1	1	1	1	0	1	0	1	0	1	1	1	0	0	0	0	0	1	0	1	0	1	0	1	00 0 0 0
**2CZ**	**2020**	ST512	154	K107	O1/O2v2					** **												** **		0	0	0	0	0	1	0	0	1	0	0	0	0	0	1	1	0	0	0	1	0	0	0	0	0	1	1	0	0	0	1	1	0	1	0	1	0	11 0 0 0
**3CZ**	**2020**	ST512	154	K107	O1/O2v2					** **												** **		0	0	0	0	0	1	0	0	1	0	0	0	0	0	1	1	0	0	0	1	0	0	0	0	0	1	1	0	0	0	1	1	0	0	0	1	0	11 0 0 0
**4CZ**	**2020**	ST307	173	K102	O1/O2v2					** **												** **		1	0	0	0	1	1	1	0	1	0	0	0	0	0	1	1	0	1	0	1	1	1	0	0	0	0	1	0	0	0	1	1	1	1	0	1	0	01 0 0 0
**5CZ**	**2020**	ST512	154	K107	O1/O2v2					** **												** **		0	0	0	0	0	1	0	0	0	0	1	0	0	1	1	1	1	0	1	0	1	0	1	1	1	0	1	0	0	0	1	1	1	0	1	0	1	00 0 0 1
**6CZ**	**2020**	ST512	154	K107	O1/O2v2					** **												** **		0	0	0	0	0	1	0	0	1	0	0	0	0	0	1	1	0	0	0	1	0	0	0	0	0	1	1	0	0	0	1	1	0	1	0	1	0	11 0 0 0
**7CZ**	**2020**	ST512	154	K107	O1/O2v2					** **												** **		0	0	0	0	0	1	0	0	1	0	0	0	0	0	1	1	0	0	0	1	0	0	0	0	0	1	1	0	0	0	1	1	0	1	0	1	0	11 0 0 0
**8CZ**	**2020**	ST512	154	K107	O1/O2v2					** **												** **		0	0	0	0	0	1	0	0	1	0	0	0	0	0	1	1	0	0	0	1	0	0	0	0	0	1	0	0	0	0	1	1	0	1	0	1	0	11 0 0 0
**9CZ**	**2020**	ST512	154	K107	O1/O2v2					** **														0	0	0	0	0	1	0	0	0	0	0	0	1	0	1	1	0	0	0	1	0	0	0	0	0	1	1	0	0	0	1	1	0	1	0	1	0	11 0 0 0
**10CZ**	**2020**	ST101	137	K17	O1/O2v1					** **														0	0	0	0	0	1	0	1	0	0	1	0	0	0	0	0	0	1	0	1	0	0	0	0	0	0	1	0	0	1	0	1	0	1	0	1	0	01 1 1 0
**12CZ**	**2021**	ST307	173	K102	O1/O2v2					** **														1	0	0	0	1	1	1	0	1	0	0	0	0	0	1	1	0	1	0	1	1	0	1	0	1	0	1	0	0	0	1	1	1	1	0	1	1	01 0 0 0

Antibiotic tested: CZA, ceftazidime/avibactam; AMC, amoxicillin/clavulanate; TZP, piperacillin/tazobactam; FEP, cefepime; CAZ, ceftazidime; C/T, ceftazolane/tazobactam; MEM- meropenem; IMI, imipenem; AZT, aztreonam; MEV- meropenem/vaporbactam ; FDC, cefiderocol; CIP, ciprofloxacin; TOB, tobramycin; AK, amikacin; CN, gentamycin; CS, colistin; FOS, fosfomycin; SXT,trimethoprim/sulfamethoxazole.

The antibiotic resitance or gene presence were reported as a coloured square:
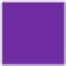
Beta-lactams,
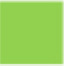
Fluorochinolones,
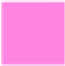
Aminoglycoside,
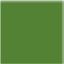
Colistine,
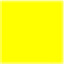
Fosfomycine,
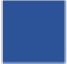
Sulfonamides,
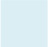
Miscellaneus.

The *bla*_CTX-M-15_ gene was detected in two isolates, while a plethora of other acquired determinants associated with resistance to beta-lactams (*bla*_OXA-1-9-48_ and *bla*_TEM-181_), aminoglycosides (*aph*, *AAC*, *aad*, *ANT* and *arm*A), sulfonamides (*sul*1 and *sul*2), trimetroprim (*dfr*A12 and *dfr*A14), quinolones (*qnr*S1 and *qnr*B17), macrolides (*mph*A and *mph*E) and phenicoles (*cat*B3 and *cat*L) were also variably present. The *fos*A6 gene, associated with fosfomycin resistance, was detected in 10/16 strains, independently linked to the MIC values of this drug. In five strains out of 16, the *arm*A gene for 16rRNA methylase was detected. Overall, the resistome was generally consistent with the resistance profile.

Remarkable differences were observed when comparing CZA, MEM and MEV susceptibility profiles with their resistome as described in **Table 3**.

**Table 3:** Phenotype Resistance and Resistome in CZA Resistant *K. pneumonia*.

					Resistance Determinat													
Samples	CZA	MEM	MEM/VAB					Beta-lactams				OmpK35^2^		OmpK36^3^		OmpK37^4^		PBP3
	S≤8 R>8	S≤2 R>2	S≤8 R>8	MLST	KPC	Reference Used by CLC analysis	SNP in KPC3^1^	SHV	OXA	TEM-181	CTX-M15	Mutation	Status	Mutation	Status	Mutation	Status	Status
**1CT**	16	1	0,5	ST101	KPC 31	Card 102956	–	SHV 28/100	–	–	–		Fs		DT ins	c. 77 ins +3	Fs	wt
**2CT**	16	16	2	ST101	KPC 34	NCBI 203501	–	–	–	–	–		Fs		DT ins	c. 77 ins +3	Fs	wt
**3CT**	16	1	0,5	ST101	KPC 28	Card 102955	–	SHV 100	–	–	–	c. 181 del G	Fs	c.408 ins +6	DT ins	c. 77 ins +3	Fs	wt
**4CT**	16	16	64	ST101	KPC 3	Card 101087	wt	SHV 187/212	OXA 48	–	–		Fs		DT ins	c. 77 ins +3	Fs	wt
**5CT**	16	2	0,25	ST101	KPC 3	NCBI 203923	c. 498 del -6 GAG; CTG	SHV 45/55	–	–	–		Fs		DT ins	c. 77 ins +3	Fs	wt
**1CZ**	16	16	0,06	ST307	KPC 3	Card 101087	wt	SHV 28/106/187/205	OXA 1/9	TEM-181	CTX-M-15	wt	wt	wt	wt	c. 77 ins +3	Fs	wt
**2CZ**	16	>32	2	ST512	KPC 3	Card 101087	wt	SHV 187	OXA 9	TEM-181	–		Fs		GD ins	c. 77 ins +3	Fs	wt
**3CZ**	16	16	0,25	ST512	KPC 3	Card 101087	wt	SHV 187	OXA 9	TEM-181	–		Fs		GD ins	c. 77 ins +3	Fs	wt
**4CZ**	16	>32	0,5	ST307	KPC 3	Card 101087	wt	SHV 28/106/187/205	OXA 9	TEM-181	–		Fs		GD ins	c. 77 ins +3	Fs	wt
**5CZ**	32	4	1	ST512	KPC 31	Card 102956	–	–	OXA 1/9	TEM-181	CTX-M15	c.119 ins G	Fs	c.405 ins +6	GD ins	c. 77 ins +3	Fs	wt
**6CZ**	16	>32	1	ST512	KPC 3	Card 101087	wt	SHV 187	OXA 9	TEM-181	–		Fs		GD ins	c. 77 ins +3	Fs	wt
**7CZ**	16	>32	1	ST512	KPC 3	Card 101087	wt	SHV 187	OXA 9	TEM-181	–		Fs		GD ins	c. 77 ins +3	Fs	wt
**8CZ**	32	>32	0,5	ST512	KPC 3	Card 101087	wt	SHV 187	OXA 9	TEM-181	–		Fs		GD ins	c. 77 ins +3	Fs	wt
**9CZ**	32	16	0,5	ST512	KPC 50	Card 102973	–	SHV 187	OXA 9	TEM-181	–		Fs		GD ins	c. 77 ins +3	Fs	wt
**10CZ**														c. 626-627 TAT > TGG	p.209 Y > W			
** **														c.671 AAC>ACC	p.224 N >T			
** **														c. 674 GGC>GAC	p.225 G > D			
** **														c. 685 CGT>AGT	p.229 R > S			
** **														c.800 TTC>TCT	p.267 F > S			
**10CZ**	32	16	0,06	ST101	KPC 31	Card 102956	–	SHV 187/212	–	–	–	c. 180 del G	Fs	c. 934 ACT>CTG	p.312 I > L	c. 77 ins +3	Fs	wt
** **														c. 958-960 CTG>ATC	p. 320 L > I			
** **														c. 1047 GAA>GAC	p. 349 E >D			
** **														c. 1051 GAT>AGC	p.351 D > S			
** **														c. 1063-1064 CGC>AAC	p.355 R > N			
**12CZ**	32	32	0,25	ST307	KPC 3	Card 101087	wt	SHV 28/106/187/205	OXA 9	TEM-181	–	c. 684 del T	Fs	c. 405 ins +3	D ins	c. 77 ins +3	Fs	wt

1. Analysis of KPC3 gene was performed by using CLC tool and take over a deletion of six nucleotide in 5-CT strain; this deletion was previously reported by by Antinori et al CMI 2020. All others strains have a wild-type (wt) KPC3.

2. wt wild-type; fs, frameshift due to point mutations (i.e. c.181 del G: position 181 has G deletion; c.119 ins: position 119 has G insertion).

3. wt wild-type, DT ins (Aspartic Acid-Threonine Insertion), GD ins (Glycine-Aspartic Acid Insertion).

4. ins+3 (Insertion of 3 nucleotide).

CZA resistance correlated with the presence of *bla*_KPC3_ variants in 6 isolates (harboring the following variants: 31, 34, 28, 50), while in one strain (5CT), a *bla*_KPC3_ variant with a deletion of two amino acids (Glu and Leu) in the 167 and 168 (498del-6 nucleotide GAG/CTG) position was found. In strain 4CT, resistant amongst others to MER/VAB, the presence of a wild type *bla*_KPC-3_ together with *bla*_OXA-48_ and *bla*_SHV_ variants was associated with a full resistance profile. The remaining strains harboring wild-type *bla*_KPC3_ showed a complex array of *bla*_SHV_ variants, together with *bla*_TEM-181_; in two strains, namely 1CZ and 5CZ, *bla*_CTX-M-15_ was found.

Membrane permeability was also investigated: in 16/17 strains *omp*K35 harbored frameshift mutations while *omp*K36 showed variants (insertions and SNPs), only in CZ1 wild type proteins were detected. With regard to *omp*K37, all strains harbored a non-functional protein. Our isolates carried wild-type PBP3 genes.

A statistical correlation analysis of all beta-lactamase genes was performed using the Pearson coefficient (**Figure S1**), which showed a direct significant association between the presence of *bla*_SHV187_ with *bla*_OXA-9_ (0.75) and *bla*_TEM-181_, between *bla*_SHV-28_ and *bla*_SHV-100_ (0.51), *bla*_SHV-105_ and *bla*_SHV-106_ (0.71), between *bla*_SHV-100_ and *bla*_KPC-28_ (0.68), with the most significant association found for *bla*_SHV-45_ and *bla*_SHV-55_, *bla*_SHV-106_ and *bla*_SHV-205_, *bla*_OXA-1_ and *bla*_CTX-M-15_, and *bla*_OXA-9_ and *bla*_TEM-181_ (1).

### Virulome analysis

The Virulence Factor Database was used to predict and compare virulence genes. All CZA-resistant isolates carried a large array of virulence determinants (**Table 4**).

**Table 4:** Virulence Profile.

						Capsule	RcsAB		rmpA2	LPS							efflux pump	Type I fimbrie										Type III fibrie								Yersiniabactin											Aerobactin					Entbactine										SalmochelineT6SS																		
	Year	MLST	wzi	K-locus	O-locus	galF	rcsA	rcsB	rmpA2	gls	kfoC	wbbM	wbbN	wbbO	wzm	wzt	arcAB	fimA	fimB	fimC	fimD	fimE	fimF	fimG	fimH	fimI	fimK	mrkA	mrkB	mrkC	mrkD	mrkF	mrkH	mrkI	mrkJ	ybtP	ybtQ	fyuA	irp1	irp2	ybtE	ybtS	ybtT	ybtU	ybtX	ybtA	iucA	iucB	iucC	iucD	iutA	entA	entB	entC	entD	entE	entF	fepA	fepB	fepC	fepD	fepGfes	ybdA	iroE	tssF	tssG	scin/tssJ	clpV/tssH	dotU/tssL	hcp/tssD	icmF/tssM	impA/tssA	KPHS_23120	tle1	tli1	vasE/tssK	vgrG/tssI	vipA/tssB	vipB/tssC
**1CT**	**2020**	ST101	137	K17	O1/O2v1		1		1		0	1	0	0	1	1												mrkB								1	1	1	1	1	1	1	1	1	1	1	1	1	1	1	1	0				1		0				1	1	1	0	0	0	0	0	1	1	0	1						
**2CT**	**2020**	ST101	137	K17	O1/O2v1		1		1		0	1	1	0	1	1																				1	1	1	1	1	1	1	1	1	1	1	1	1	1	1	1	0				1		0				1	1	1	0	0	0	0	0	1	1	0	1						
**3CT**	**2020**	ST101	137	K17	O1/O2v1		1		1		0	1	1	0	1	1																				1	1	1	1	1	1	1	1	1	1	1	1	1	1	1	1	0				1		0				1	1	1	0	0	0	0	0	1	1	0	1						
**4CT**	**2021**	ST101	137	K17	O1/O2v1		1		1		0	1	1	0	1	1																				1	1	1	1	1	1	1	1	1	1	1	1	1	1	1	1	0				1		0				1	1	1	0	0	0	0	0	1	1	0	1						
**5CT**	**2021**	ST101	137	K17	O1/O2v1		0		0		0	0	0	1	0	1																				1	1	1	1	1	1	1	1	1	1	1	1	1	1	1	0	0				1		0				0	0	0	0	0	0	0	0	0	0	0	0	0					
**1CZ**	**2020**	ST307	173	K102	O1/O2v2		1		0		1	0	1	1	1	0																				1	1	1	1	1	1	0	1	1	0	0	0	0	0	0	0	1				1		1				1	1	1	0	1	1	0	0	1	1	1	1						
**2CZ**	**2020**	ST512	154	K107	O1/O2v2		1		0		1	0	1	1	1	0																				0	0	0	0	0	0	0	0	0	0	0	0	0	0	0	0	1				0		0				1	1	1	1	1	1	1	0	1	1	1	1						
**3CZ**	**2020**	ST512	154	K107	O1/O2v2		1		0		1	0	1	1	1	0																				0	0	0	0	0	0	0	0	0	0	0	0	0	0	0	0	1				0		0				1	1	1	1	1	1	1	0	1	1	1	1						
**4CZ**	**2020**	ST307	173	K102	O1/O2v2		1		0		1	0	1	1	1	0																				1	1	1	1	1	1	1	1	1	1	0	0	0	0	0	0	1				1		1				1	1	1	0	1	1	0	0	1	1	1	1						
**5CZ**	**2020**	ST512	154	K107	O1/O2v2		1		0		1	0	1	1	1	0																				0	0	0	0	0	0	0	0	0	0	0	0	0	0	0	0	1				0		0				1	1	1	1	1	1	1	0	1	1	1	1						
**6CZ**	**2020**	ST512	154	K107	O1/O2v2		1		0		1	0	1	1	1	0																				0	0	0	0	0	0	0	0	0	0	0	0	0	0	0	0	1				0		0				1	1	1	1	1	1	1	0	1	1	1	1						
**7CZ**	**2020**	ST512	154	K107	O1/O2v2		1		0		1	0	1	1	1	0																				0	0	0	0	0	0	0	0	0	0	0	0	0	0	0	0	1				0		0				1	1	1	1	1	1	1	0	1	1	1	1						
**8CZ**	**2020**	ST512	154	K107	O1/O2v2		1		0		1	0	1	1	1	0																				0	0	0	0	0	0	0	0	0	0	0	0	0	0	0	0	1				0		0				1	1	1	1	1	1	1	1	1	0	1	1						
**9CZ**	**2020**	ST512	154	K107	O1/O2v2		1		0		1	0	1	1	1	0																				0	0	0	0	0	0	0	0	0	0	0	0	0	0	0	0	1				0		0				1	1	1	1	1	1	1	0	1	1	1	1						
**10CZ**	**2020**	ST101	137	K17	O1/O2v1		1		0		0	1	1	0	1	1																				1	1	1	1	1	1	1	1	1	1	1	0	0	0	0	0	0				1		0				1	1	1	0	0	0	0	0	1	0	1	1						
**12CZ**	**2021**	ST307	173	K102	O1/O2v2		1		0		1	0	1	1	1	0																				1	1	1	1	1	1	1	1	1	1	1	0	0	0	0	0	1				1		1				1	1	1	0	1	1	0	0	1	1	1	1						

The presence of virulence genes were reported as coloured square:

- Gene associated to capsule:
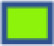
capsule production;
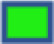
rcsAB - regulation of capsule sintesys and rmpA2 – regulation of mucoid phenotype; 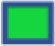
LPS;
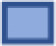
efflux pump;

- Gene associated to fibrie:
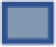
type I;
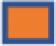
type III.

- Gene associated to syderophores:
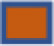
Yersiniabactin (Iron carrier, growth and replication of bacteria);
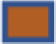
Aerobactin (Iron carrier, growth and replication of bacteria, highly virulet pneumoniae);
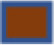
Enterobactine (Iron carrier, growth and replication of bacteria);
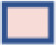
Salmocheline.

- Gene associeted to secretion system:
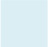
T6SS (type VI Secretion System).

As regards the genes involved in capsule production, we found that *gal*F and the genes responsible for the upregulation of capsule production (*rcs*AB system) were present in the whole sample (*rcs*A alone in 93,75% (15/16)); *rpm*A2, that regulates the mucoid phenotype, in 25% (4/16) only.

Genes for LPS synthesis were variably present: *glf* in 100%, *wzm* in 93,75% (15/16), *wbb*N-O in 68,75% (11/16), *kfo*C in 62,5% (10/16), *wzt* in 37,5 (6/16), and *wbb*M in 31,25% (5/16) of samples. The efflux pump *arc*AB gene was present in the whole sample. Furthermore, a complete set of genes for type I (*fim*A-K) and type III (*mrk*A-B-C-D-F-H-I-J) fimbriae was found in all isolates.

Genes for siderophores were variably present in our sample. The Yersiniabactin genes were found as follows: *ybt*E-P-Q-T-U, *fyu*A, *irp*1-2 in 56,25% (9/16), *ybt*S-X in 50% (8/16) and *ybt*A in 37,5% (6/16) of samples, respectively. Aerobactine genes *iuc*A-B-C were detected in 31,2% (5/16) of strains and *iut*A in 25% (4/16). Among enterobactine genes, all strains (100%) carried the e*nt*B-C-D-F, *fep*B-C-D-G, *fes* and *ybd*A loci; *ent*A was found in 62,5% (10/16), *ent*E in 56,25% (9/16) and *fep*A in 18,75% (3/16). The *iro*E gene encoding salmochelin was present in the whole sample.

The *arc*AB, *tss*F-G, *sci*N/*tss*J type VI secretion system genes, which were part of the structural core, were present in 100% of the sample, followed by *clp*V/*tss*H, *dot*U/*tss*L, *hcp*/*tss*D, *vas*E/*tss*K, *vip*A/*tss*B, *vip*B/*tss*C, found in 93,75% (15/16), *imp*A/*tss*A and KPHS_23120 in 62,5% (10/16), *vgr*G/*tss*I in 56,25% (9/16), *icm*F/*tss*M in 43,75 (7/16), and *tli*1 only in 6,25% (1/16).

## Discussion

In this study, we reported the occurrence of CZA resistance in a group of KPC-producing *K. pneumoniae* isolates collected in three hospitals located in two southern Italian regions during the pandemic period. All isolates represented different genotypes and were further analyzed for their virulome as well as for the consistency of their susceptibility profiles and resistome. Tracking their dissemination and understanding their evolution is an important step towards monitoring and controlling these pathogens.

Several findings are particularly noteworthy.

First of all, CZA-resistant strains showed a comparable MEM resistance profile (13/16 isolates with a MIC range of 4->32 mg/L), whereas MEV exhibited excellent *in vitro* activity against 15/16 isolates (MIC range 0.006-0.5 mg/L); full susceptibility to cefiderocol (MIC range 0.006-0.5 mg/L) was also observed.

MDR susceptibility profile included resistance to aminoglycosides and fluoroquinolones; 9 strains were also resistant to colistin and 7/16 to fosfomycin, in the absence of strict correlation with the presence of the *fos*A gene, which was found in all ST512 and ST307 isolates, but not in ST101.

Remarkable differences were observed when analyzing consistency of the CZA, MEM and MEV phenotypic profiles with the corresponding resistome. CZA resistance correlated with the presence of *bla*_KPC3_ variants in 6 isolates, in which different alleles were found (namely *bla*_KPC31-34-28-50_). One strain (5CT) harbored a *bla*_KPC-3_ variant with a deletion of two amino acids (Glu and Leu) in the 167 and 168 (498del-6 nucleotide GAG/CTG) position, as previously described (Antinori et al., 2020). All other carried *bla*_KPC-3_ wild-type genes variably associated with a plethora of other resistance determinants; among these, one strain (4CT) carried *bla*_KPC-3_ with *bla*_OXA-48_ and *bla*_SHV-187_, associated to MEV resistance.

The remaining *bla*_KPC-3_ carriers carried different alleles for *bla*_SHV_, *bla*_TEM_ and *bla*_OXA_. Strains 1 and 5CZ also carried *bla*_CTX-M-15_.

Even though non-carbapenemase beta-lactamases, such as ESBLs, have the capacity to hydrolyze carbapenemase (Paterson and Bonomo, 2005), in our study we were not able to determine their contribution, suggesting – as already observed (Paterson and Bonomo, 2005) – that their presence did not increase carbapenemase resistance among isolates that already harbored a carbapenemase gene.

Adding to this complex gene acquisition, all isolates have mutation in OmpK35, OmpK36 and OmpK37, related to deletion, insertion and frameshift.

In our study, we confirmed that, in KPC producing-*K. pneumoniae* strains, mutations in the porine gene *omp*K36 were present and, as previously observed by other Authors (Venditti et al., 2021) presumably correlate with high-level resistance to carbapenems (Wong et al., 2019; David et al., 2022). In addition to the indel found in the *omp*K36 gene, our isolates harbored frameshift mutations in *omp*K35 and *omp*K37, leading to nonfunctional proteins, associated, also in this case, with lower carbapenemase MICs, as already described by other Authors (Gaibani et al., 2020; Venditti et al., 2021).

Outer membrane protein alterations can also influence carbapenem activity in *K. pneumoniae*; in fact, loss of function of one or multiple porin channels can diminish their activity.

Even though we could not identify the role of each mutation in our study, our data support the role of several biologically plausible genotypes in the determination of resistance. Interestingly, in our isolates vaborbactam maintained its ability to enter the bacterial cell despite the loss of the two major proteins OmpK35 and OmpK37. It is already known that vaborbactam crosses the outer membrane of *K. pneumoniae* exploiting both OmpK35 and OmpK36, with the latter being the preferred one (Venditti et al., 2021). In our isolates, the mutations found in OmpK36 were unable to affect the entry ability of the inhibitor, thus reducing the MIC values of meropenem. Generally speaking, despite this complexity, MEV (15/16 isolates) and cefiderocol were still in the range of susceptibility in all our CZA resistant isolates, proving able to maintain reliable *in vitro* activity against these difficult-to-treat isolates. Taken together, these data showed that a new cephalosporin with a new mechanism of entry, and vaborbactam a new beta lactamase inhibitor (BLI), can inhibit these isolates.

Finally, the 16 isolates belonged to three different STs, namely ST101, ST307 and ST512, each associated with a specific capsular locus (17, 102 and 107) and an O-antigen. ST101, found only in Catania and in one strain from Catanzaro, was associated to the O1/O2v1 variant, while both ST307 and ST512 have somatic antigen variants, i.e., O1/O2v1 and O1/O2v2 (Artyszuk et al., 2020). Of interest in these isolates is the co-presence of the *rmp*A2 regulator and the *iut*A aerobactin in 4 strains from Catania, indicating a hypermucoviscosity phenotype (Paczosa and Mecsas, 2016; Wang et al., 2020). Furthermore, all ST101 isolates carried the *wzi* gene, encoding the surface protein involved in capsule attachment to the outer membrane (Gona et al., 2019).

## Conclusion

In conclusion, CZA has definitely become an important first-line option for KPC-*Kp* infections. With this increased use, the expected resistance will continue to emerge, both through the spread of high-level epidemic clones and the dissemination of plasmids carrying mutant genes.

The results of this study provide further evidence of the plasticity and evolutionary potential of CAZ and MER-resistant clones of *K. pneumoniae*, showing evidence of multiple adaptation and raising concerns about the strong selective pressure acting on all drugs, including novel carbapenemase inhibitors.

The convergence of clinically relevant resistance and virulence determinants, including hypervirulent clones, may enable the parallel evolution of resistance and virulence, which is a worrisome event.

With the increasing number of MDR lineages harboring variants of resistance determinants, it becomes mandatory to detect and track their dissemination by using high throughput technologies.

Further studies are urgently needed to validate the efficacy of new drugs in case of CZA resistance, under the guide of advanced molecular resistance tests in conjunction with phenotypic results and eventually synergy analyses.

## Data availability statement

The datasets presented in this study can be found in online repositories. The names of the repository/repositories and accession number(s) can be found at: http://www.ncbi.nlm.nih.gov/bioproject/PRJNA866305.

## Author contributions

The first author named is lead and corresponding author. We describe contributions to the paper using the taxonomy provided above. Writing – Original Draft: DB, DAB, MM, GP, and SS. Writing – Review and Editing: GM, CT, and SS. Conceptualization: GM, CT, SS, and MM. Investigation: DAB and CC. Methodology: DB, ET, AR, NM, and AQ. Software: NiM and GP. Formal Analysis: NiM, DB, and DAB. Validation: ET, AR, NM, AQ, and GS. Visualization: DB, NM, MM, AQ, and GS. Project Administration, Funding Acquisition and Resouces: GM, CT, and SS. All authors contributed to the article and approved the submitted version.
