# Supplementary material for: Omic insights into various ceftazidime-avibactam-resistant Klebsiella pneumoniae isolates from two southern Italian regions
Source: Front Cell Infect Microbiol. 2023 Jan 5;12:1010979. doi: 10.3389/fcimb.2022.1010979 (PMC9851273; doi:10.3389/fcimb.2022.1010979)
Supplement: Supplementary file 1 [file Presentation_1.pptx]

## Slide 1
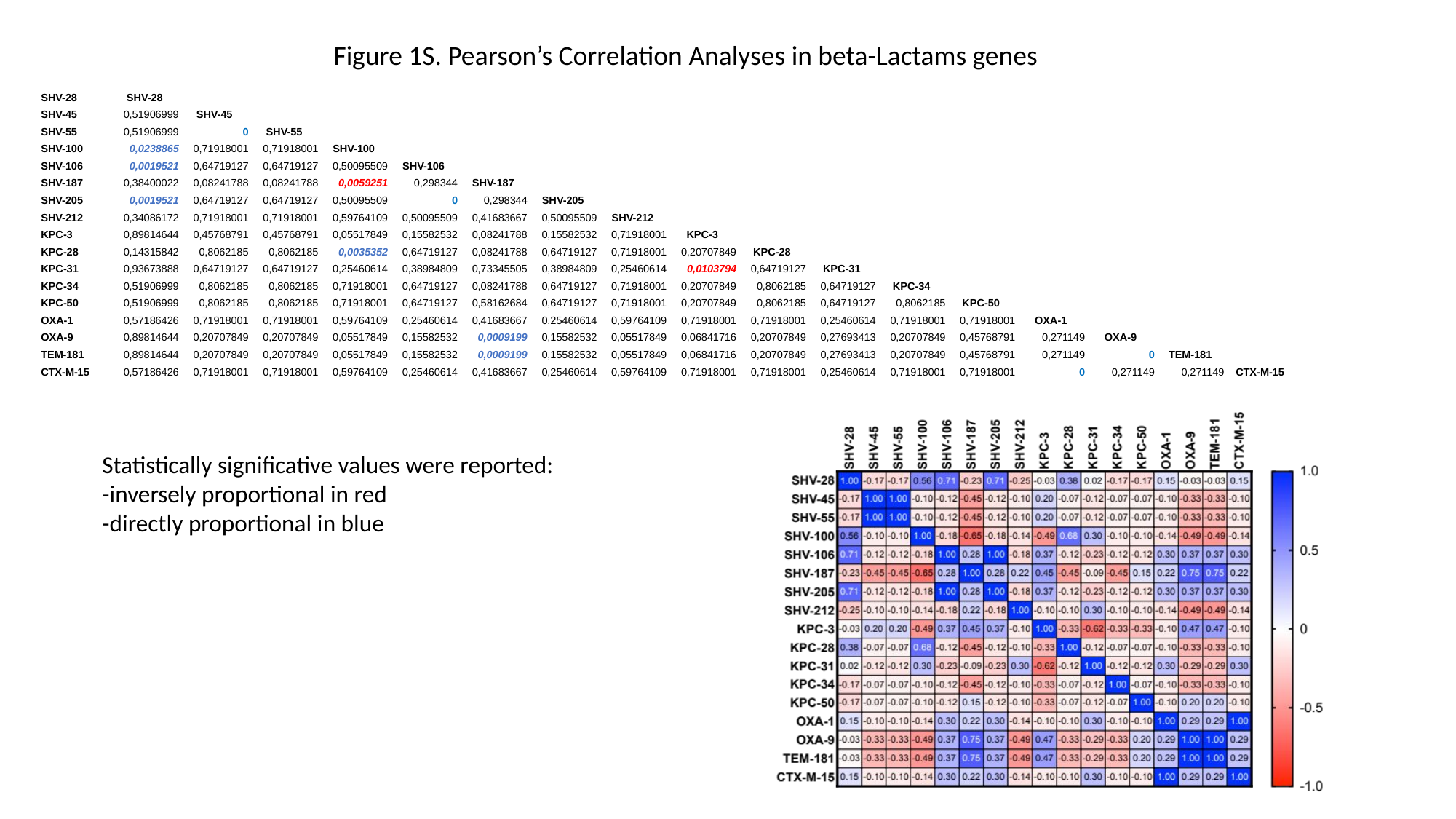

Figure 1S. Pearson’s Correlation Analyses in beta-Lactams genes
| SHV-28 | SHV-28 | | | | | | | | | | | | | | | | |
| --- | --- | --- | --- | --- | --- | --- | --- | --- | --- | --- | --- | --- | --- | --- | --- | --- | --- |
| SHV-45 | 0,51906999 | SHV-45 | | | | | | | | | | | | | | | |
| SHV-55 | 0,51906999 | 0 | SHV-55 | | | | | | | | | | | | | | |
| SHV-100 | 0,0238865 | 0,71918001 | 0,71918001 | SHV-100 | | | | | | | | | | | | | |
| SHV-106 | 0,0019521 | 0,64719127 | 0,64719127 | 0,50095509 | SHV-106 | | | | | | | | | | | | |
| SHV-187 | 0,38400022 | 0,08241788 | 0,08241788 | 0,0059251 | 0,298344 | SHV-187 | | | | | | | | | | | |
| SHV-205 | 0,0019521 | 0,64719127 | 0,64719127 | 0,50095509 | 0 | 0,298344 | SHV-205 | | | | | | | | | | |
| SHV-212 | 0,34086172 | 0,71918001 | 0,71918001 | 0,59764109 | 0,50095509 | 0,41683667 | 0,50095509 | SHV-212 | | | | | | | | | |
| KPC-3 | 0,89814644 | 0,45768791 | 0,45768791 | 0,05517849 | 0,15582532 | 0,08241788 | 0,15582532 | 0,71918001 | KPC-3 | | | | | | | | |
| KPC-28 | 0,14315842 | 0,8062185 | 0,8062185 | 0,0035352 | 0,64719127 | 0,08241788 | 0,64719127 | 0,71918001 | 0,20707849 | KPC-28 | | | | | | | |
| KPC-31 | 0,93673888 | 0,64719127 | 0,64719127 | 0,25460614 | 0,38984809 | 0,73345505 | 0,38984809 | 0,25460614 | 0,0103794 | 0,64719127 | KPC-31 | | | | | | |
| KPC-34 | 0,51906999 | 0,8062185 | 0,8062185 | 0,71918001 | 0,64719127 | 0,08241788 | 0,64719127 | 0,71918001 | 0,20707849 | 0,8062185 | 0,64719127 | KPC-34 | | | | | |
| KPC-50 | 0,51906999 | 0,8062185 | 0,8062185 | 0,71918001 | 0,64719127 | 0,58162684 | 0,64719127 | 0,71918001 | 0,20707849 | 0,8062185 | 0,64719127 | 0,8062185 | KPC-50 | | | | |
| OXA-1 | 0,57186426 | 0,71918001 | 0,71918001 | 0,59764109 | 0,25460614 | 0,41683667 | 0,25460614 | 0,59764109 | 0,71918001 | 0,71918001 | 0,25460614 | 0,71918001 | 0,71918001 | OXA-1 | | | |
| OXA-9 | 0,89814644 | 0,20707849 | 0,20707849 | 0,05517849 | 0,15582532 | 0,0009199 | 0,15582532 | 0,05517849 | 0,06841716 | 0,20707849 | 0,27693413 | 0,20707849 | 0,45768791 | 0,271149 | OXA-9 | | |
| TEM-181 | 0,89814644 | 0,20707849 | 0,20707849 | 0,05517849 | 0,15582532 | 0,0009199 | 0,15582532 | 0,05517849 | 0,06841716 | 0,20707849 | 0,27693413 | 0,20707849 | 0,45768791 | 0,271149 | 0 | TEM-181 | |
| CTX-M-15 | 0,57186426 | 0,71918001 | 0,71918001 | 0,59764109 | 0,25460614 | 0,41683667 | 0,25460614 | 0,59764109 | 0,71918001 | 0,71918001 | 0,25460614 | 0,71918001 | 0,71918001 | 0 | 0,271149 | 0,271149 | CTX-M-15 |
Statistically significative values were reported:
-inversely proportional in red
-directly proportional in blue

## Slide 2
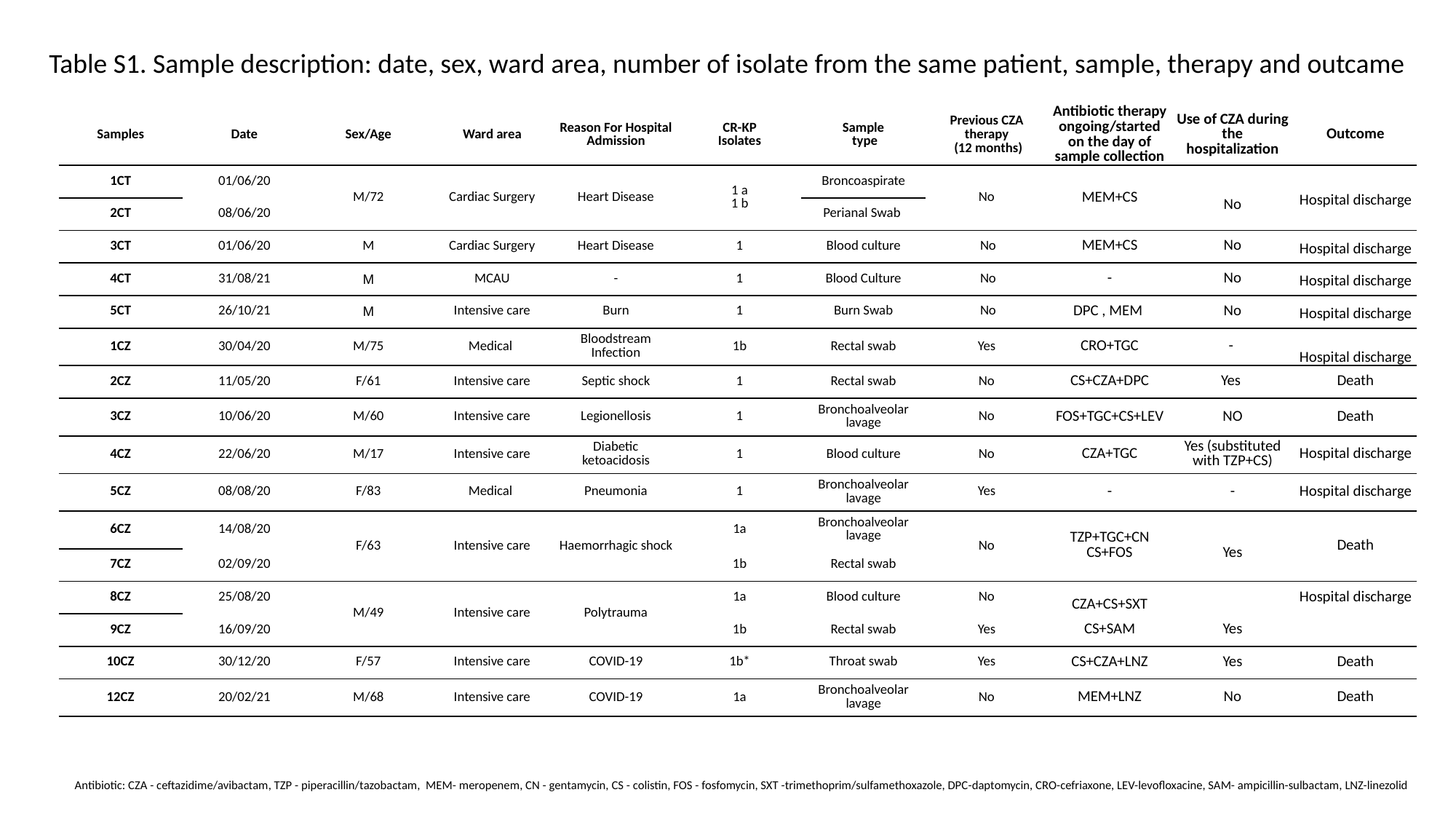

Table S1. Sample description: date, sex, ward area, number of isolate from the same patient, sample, therapy and outcame
| Samples | Date | Sex/Age | Ward area | Reason For Hospital Admission | CR-KP Isolates | Sample type | Previous CZA therapy (12 months) | Antibiotic therapy ongoing/started on the day of sample collection | Use of CZA during the hospitalization | Outcome |
| --- | --- | --- | --- | --- | --- | --- | --- | --- | --- | --- |
| 1CT | 01/06/20 | M/72 | Cardiac Surgery | Heart Disease | 1 a 1 b | Broncoaspirate | No | MEM+CS | No | Hospital discharge |
| 2CT | 08/06/20 | | | | | Perianal Swab | | | | |
| 3CT | 01/06/20 | M | Cardiac Surgery | Heart Disease | 1 | Blood culture | No | MEM+CS | No | Hospital discharge |
| 4CT | 31/08/21 | M | MCAU | - | 1 | Blood Culture | No | - | No | Hospital discharge |
| 5CT | 26/10/21 | M | Intensive care | Burn | 1 | Burn Swab | No | DPC , MEM | No | Hospital discharge |
| 1CZ | 30/04/20 | M/75 | Medical | Bloodstream Infection | 1b | Rectal swab | Yes | CRO+TGC | - | Hospital discharge |
| 2CZ | 11/05/20 | F/61 | Intensive care | Septic shock | 1 | Rectal swab | No | CS+CZA+DPC | Yes | Death |
| 3CZ | 10/06/20 | M/60 | Intensive care | Legionellosis | 1 | Bronchoalveolar lavage | No | FOS+TGC+CS+LEV | NO | Death |
| 4CZ | 22/06/20 | M/17 | Intensive care | Diabetic ketoacidosis | 1 | Blood culture | No | CZA+TGC | Yes (substituted with TZP+CS) | Hospital discharge |
| 5CZ | 08/08/20 | F/83 | Medical | Pneumonia | 1 | Bronchoalveolar lavage | Yes | - | - | Hospital discharge |
| 6CZ | 14/08/20 | F/63 | Intensive care | Haemorrhagic shock | 1a | Bronchoalveolar lavage | No | TZP+TGC+CN CS+FOS | Yes | Death |
| 7CZ | 02/09/20 | | | | 1b | Rectal swab | | | | |
| 8CZ | 25/08/20 | M/49 | Intensive care | Polytrauma | 1a | Blood culture | No | CZA+CS+SXT | | Hospital discharge |
| 9CZ | 16/09/20 | | | | 1b | Rectal swab | Yes | CS+SAM | Yes | |
| 10CZ | 30/12/20 | F/57 | Intensive care | COVID-19 | 1b\* | Throat swab | Yes | CS+CZA+LNZ | Yes | Death |
| 12CZ | 20/02/21 | M/68 | Intensive care | COVID-19 | 1a | Bronchoalveolar lavage | No | MEM+LNZ | No | Death |
Antibiotic: CZA - ceftazidime/avibactam, TZP - piperacillin/tazobactam, MEM- meropenem, CN - gentamycin, CS - colistin, FOS - fosfomycin, SXT -trimethoprim/sulfamethoxazole, DPC-daptomycin, CRO-cefriaxone, LEV-levofloxacine, SAM- ampicillin-sulbactam, LNZ-linezolid

## Slide 3
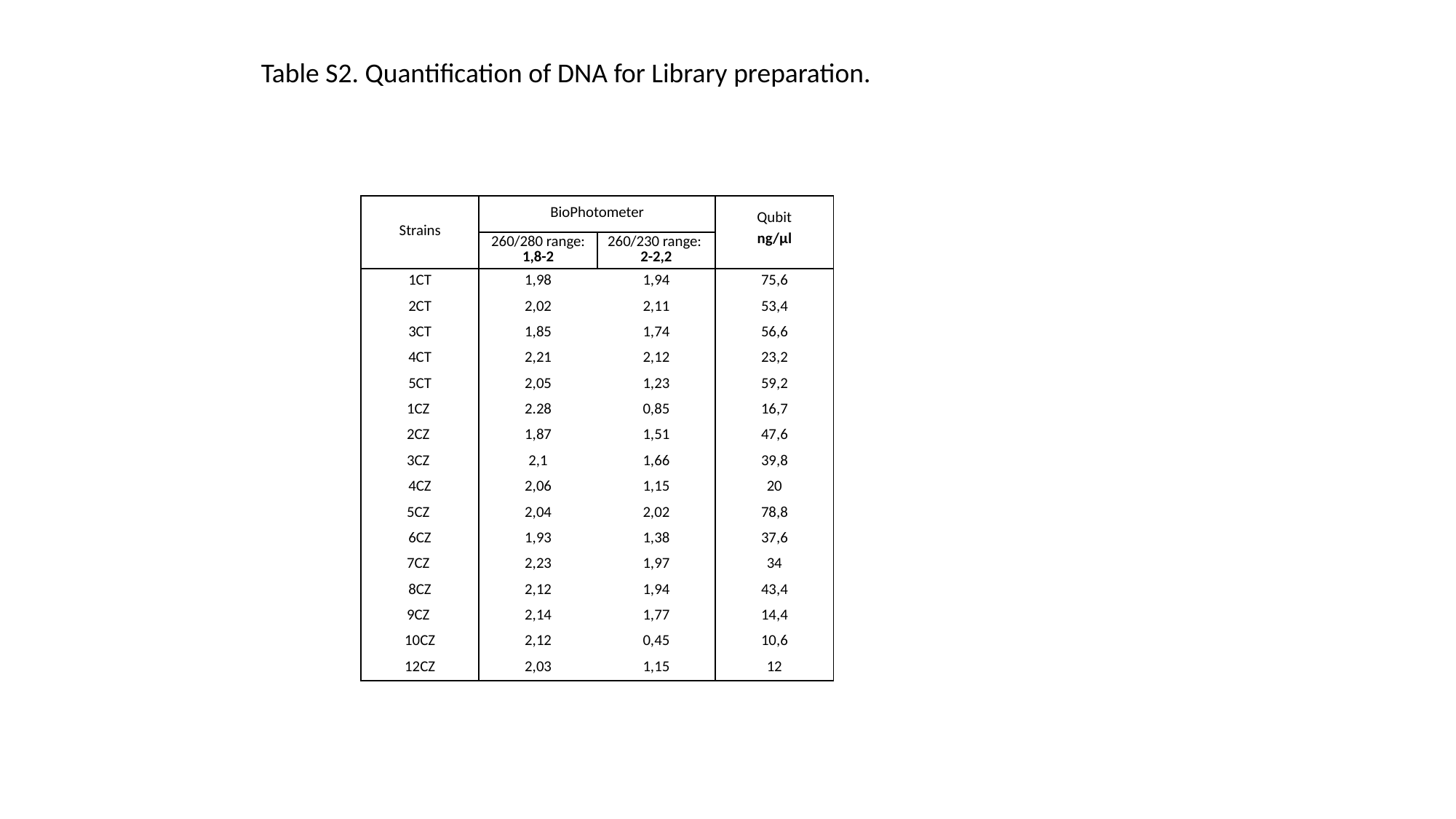

Table S2. Quantification of DNA for Library preparation.
| Strains | BioPhotometer | | Qubit |
| --- | --- | --- | --- |
| | 260/280 range: 1,8-2 | 260/230 range: 2-2,2 | ng/µl |
| 1CT | 1,98 | 1,94 | 75,6 |
| 2CT | 2,02 | 2,11 | 53,4 |
| 3CT | 1,85 | 1,74 | 56,6 |
| 4CT | 2,21 | 2,12 | 23,2 |
| 5CT | 2,05 | 1,23 | 59,2 |
| 1CZ | 2.28 | 0,85 | 16,7 |
| 2CZ | 1,87 | 1,51 | 47,6 |
| 3CZ | 2,1 | 1,66 | 39,8 |
| 4CZ | 2,06 | 1,15 | 20 |
| 5CZ | 2,04 | 2,02 | 78,8 |
| 6CZ | 1,93 | 1,38 | 37,6 |
| 7CZ | 2,23 | 1,97 | 34 |
| 8CZ | 2,12 | 1,94 | 43,4 |
| 9CZ | 2,14 | 1,77 | 14,4 |
| 10CZ | 2,12 | 0,45 | 10,6 |
| 12CZ | 2,03 | 1,15 | 12 |

## Slide 4
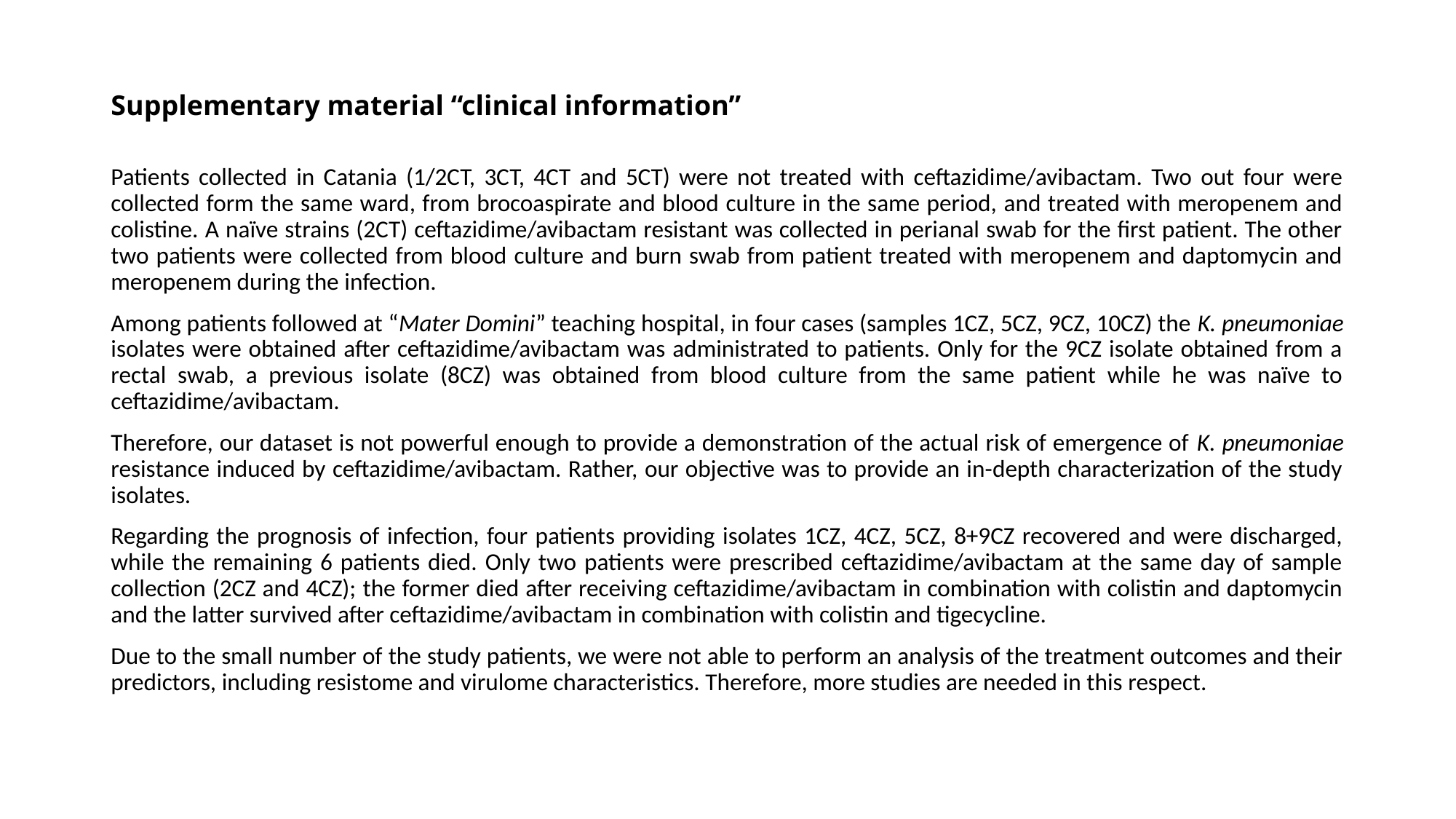

# Supplementary material “clinical information”
Patients collected in Catania (1/2CT, 3CT, 4CT and 5CT) were not treated with ceftazidime/avibactam. Two out four were collected form the same ward, from brocoaspirate and blood culture in the same period, and treated with meropenem and colistine. A naïve strains (2CT) ceftazidime/avibactam resistant was collected in perianal swab for the first patient. The other two patients were collected from blood culture and burn swab from patient treated with meropenem and daptomycin and meropenem during the infection.
Among patients followed at “Mater Domini” teaching hospital, in four cases (samples 1CZ, 5CZ, 9CZ, 10CZ) the K. pneumoniae isolates were obtained after ceftazidime/avibactam was administrated to patients. Only for the 9CZ isolate obtained from a rectal swab, a previous isolate (8CZ) was obtained from blood culture from the same patient while he was naïve to ceftazidime/avibactam.
Therefore, our dataset is not powerful enough to provide a demonstration of the actual risk of emergence of K. pneumoniae resistance induced by ceftazidime/avibactam. Rather, our objective was to provide an in-depth characterization of the study isolates.
Regarding the prognosis of infection, four patients providing isolates 1CZ, 4CZ, 5CZ, 8+9CZ recovered and were discharged, while the remaining 6 patients died. Only two patients were prescribed ceftazidime/avibactam at the same day of sample collection (2CZ and 4CZ); the former died after receiving ceftazidime/avibactam in combination with colistin and daptomycin and the latter survived after ceftazidime/avibactam in combination with colistin and tigecycline.
Due to the small number of the study patients, we were not able to perform an analysis of the treatment outcomes and their predictors, including resistome and virulome characteristics. Therefore, more studies are needed in this respect.
